# Exploratory Analysis of Gut Microbiota Profile in Duchenne Muscular Dystrophy (DMD) Patients with Intellectual Disability

**DOI:** 10.1007/s12035-025-04974-7

**Published:** 2025-05-05

**Authors:** Chiara Panicucci, Sara Casalini, Giovanni Fiorito, Alessandra Biolcati Rinaldi, Valentina Biagioli, Davide Cangelosi, Noemi Brolatti, Elisa Principi, Serena Baratto, Marina Pedemonte, Simone Morando, Antonella Riva, Cristina Venturino, Pasquale Striano, Paolo Uva, Claudio Bruno

**Affiliations:** 1 Centre of Translational and Experimental Myology, IRCCS Istituto Giannina Gaslini, Genoa, Italy; 2 Clinical Bioinformatics Unit, IRCCS Istituto Giannina Gaslini, Genoa, Italy; 3 Clinical Psychology Unit, IRCCS Istituto Giannina Gaslini, Genoa, Italy; 4 Pediatric Neurology and Muscle Diseases Unit, IRCCS Istituto Giannina Gaslini, Genoa, Italy; 5 Department of Neurosciences, Rehabilitation, Ophthalmology, Genetics, Maternal and Child Health (DINOGMI), University of Genova, Genoa, Italy

**Keywords:** Duchenne muscular dystrophy, Intellectual disability, Gut-brain axis, Metagenomics, 16S rRNA gene sequencing

## Abstract

This study investigates the differences in gut microbiota composition between DMD patients with (DMD +) and without (DMD −) intellectual disability (ID) and its potential role in cognitive outcomes. In this study, we assessed the gut microbiota in 50 genetically confirmed DMD patients (median age 13.1 years) using 16S rRNA gene sequencing. Cognitive assessment was performed using the Wechsler Intelligence Scales, with ID defined as an IQ < 70. Stool samples were analyzed, and statistical methods were used to assess alpha- and beta-diversity. Thirty-four percent of patients had ID. No significant differences were found in alpha-diversity or in the *Firmicutes/Bacteroidetes* ratio. However, beta-diversity analysis revealed significant differences between DMD + and DMD − groups, including, in DMD + , an increased abundance of *Propionibacterium* and *Bifidobacterium*, and a reduction in *Bulleidia*. These bacteria are involved in metabolic pathways that can influence neurological health through the gut-brain axis, particularly via the production of short-chain fatty acids. While these preliminary findings suggest a possible association between gut microbiota profile and cognitive impairment in DMD, further research is needed to explore a causal relationship and consider microbiota-targeted therapeutic strategies.

**Supplementary Information:**

The online version contains supplementary material available at 10.1007/s12035-025-04974-7.

## Introduction

Duchenne muscular dystrophy (DMD) is an X-linked disorder caused by mutations in the dystrophin (DMD) gene, characterized by progressive muscle strength decline, leading to loss of independent mobility during adolescence, along with respiratory insufficiency and dilated cardiomyopathy [1].

Alternative splicing of the DMD gene produces several isoforms of dystrophin (full-length Dp427, and shorter isoforms Dp260, Dp140, Dp116, Dp71, and Dp40), with Dp140 and Dp71 abundantly expressed in the brain, while Dp260 and Dp116 are limited to the retina and peripheral nerves, respectively [2]. Mutations at the 3′ end of the DMD gene, which affect all DMD products, have been associated with a high prevalence of brain-related comorbidities [3, 4], impacting the cognitive domain, with intellectual disability (ID), difficulties in working memory and executive functions, the learning domain, the psychiatric domain, with conditions such as attention deficit hyperactivity disorder (ADHD), autism spectrum disorder (ASD), and obsessive compulsive disorder (OCD), and the emotional domain, including anxiety and depression [3–13]. ID is reported in a significant proportion of individuals with DMD, ranging from 20 to 50%, and intellectual performance is one standard deviation (SD) below the average [7, 10–14].

The gut-brain axis, a bidirectional communication pathway between the brain and the gut, has been identified as an important modulator of several neurological and psychiatric disorders, primarily through the function of the gut microbiota [15, 16]. The gut microbiota consists of microorganisms residing in the human intestine and plays a role in metabolic, immunological, and neurological functions, mainly by the secretion of short chain fatty acids (SCFAs) [17]. SCFAs are bacterial products derived from fermentation of dietary carbohydrates, odd-chain fatty acids, and some proteins in the gastrointestinal tract [18–20]. From the gut lumen, SCFAs reach the bloodstream and, through the blood–brain barrier (BBB), reach the central nervous system (CNS), where they are involved in several functions, including BBB regulation, neurotransmitter release, and microglia regulation [21, 22].

The proper functioning of the gut microbiota depends on maintaining a stable composition, which, in humans, is mostly composed of two dominant phyla, *Bacteroidetes* and *Firmicutes*, that account for 90% of the total community [23]. An imbalance in their ratio, a reduction of microbial diversity, or the overgrowth of other groups can result in dysbiosis [24, 25]. This dysregulation can affect brain function and contribute to neurological disorders such as epilepsy, autism, Alzheimer’s and Parkinson’s disease, becoming a potential therapeutic target [16, 26–29].

In *mdx* mice, the most widely used mouse model for DMD, the genetic analysis of gut microbiota (gut metagenomics) has been recently explored in relation to muscle function (the gut-muscle axis) [30–34], revealing a significant dysbiosis compared to wild-type animals, and identifying the gut microbiota as a target to modulate muscular inflammatory, fibrotic, and autophagic processes, possibly through modulation of SCFAs. Besides these results, no data are available on the connection between gut microbiota and cognitive outcomes, neither in mice nor in DMD patients.

The aim of this work is to evaluate the gut metagenomics profile using 16S rRNA analysis in DMD patients with (DMD +) and without ID (DMD −), to uncover potential associations between abnormalities in gut microbiota and intellectual outcomes.

## Materials and Methods

### Inclusion and Exclusion Criteria and Clinical Assessments

This observational, cross-sectional study enrolled genetically confirmed DMD patients referred to the Neuromuscular Unit at Giannina Gaslini Hospital, Genoa, between May 2021 and September 2022, and selected according to the following inclusion criteria: (i) genetic diagnosis of DMD and (ii) willingness to perform neuropsychological assessments. Exclusion criteria were (i) consumption of antibiotics or immunosuppressive treatments other than steroids in the 3 months before enrollment, (ii) consumption of probiotics in the 2 weeks before enrollment, (iii) patients on a selective diet, and (iv) concomitant chronic inflammatory bowel disease.

### Study Assessments

At the enrolment visit, anamnestic data, such as age at steroid treatment initiation, age at loss of ambulation, and age at non-invasive ventilation (NIV) initiation, were collected by charts review; anthropometric parameters (height and weight) were measured, and body mass index standard deviation score (BMI SDS) was calculated according to the Italian growth charts [35].

Genetic mutations were categorized into four groups based on their locations, to predict their effect on dystrophin isoforms deficiency, as previously described [6]: group 1, mutations upstream of intron 44 inclusive (Dp427 absent, Dp140/Dp71 present); group 2, mutations located between exons 51 and 62 inclusive (Dp427/Dp140 absent, Dp71 present); and group 3, mutations downstream of exon 63 inclusive (Dp427/Dp140/Dp71 absent). Participants with mutations located between exons 45 and 50 inclusive and not involving exon 51 or located downstream exon 51 were not considered for this analysis because of the uncertainty in predicting their effects on the Dp140 DMD isoform (the Dp140 promoter is located in intron 44, while its translation initiation site lies in exon 51).

Intellectual function was assessed by two child psychologists (ABR and CV), with different intelligence scales, according to patient age, namely (i) the Wechsler Preschool and Primary Scale of Intelligence-III (WPPSI-III) for patients aged between 2 and 6 years, (ii) the Wechsler Intelligence Scale for Children-IV (WISC-IV) for boys aged between 6 and 16 years and 11 months, (ii) the Wechsler Adult Intelligent Scale-IV (WAIS-IV) for patients older than 17 years. Each scale allowed calculation of the full scale intelligence quotient (FSIQ) score. Intellectual disability was defined as FSIQ values < 70 (DMD + group), according to the DSM IV-TR. Patients with FSIQ score > 70 were categorized in the group without intellectual disability (DMD −).

For some patients, logistical constraints prevented the administration of neuropsychological scales (outpatients who could not perform complete neuropsychological assessments within the available time frame due to logistical factors and scheduling constraints). In these instances, intellectual assessment was based on their school performance [36, 37]: patients who could attend school independently, without the aid of a support teacher, were categorized in the DMD − group, while those who required assistance or significant curricular adaptations, were classified into the DMD + group.

At the enrolment visit, a stool sample was collected in a collection tube (CeGaT, Tübingen, Germany) to perform the 16S ribosomal RNA (rRNA) gene sequencing. Concurrently, patients or caregivers were administered with the Bristol stool form scale (BSFS), a validated tool to assess stool form, which classifies human feces into seven consistency categories, with lower scores associated with hard stools and longer colon transit times, and highest scores corresponding to loose stools and fast transit [38, 39].

Participants or caregivers completed a nutritional questionnaire assessing the weekly consumption frequency of macronutrients such as proteins (red meat and ham, white meat and fish, eggs, cheese, and yogurt), carbohydrates (white flour, whole flour, fruits), vegetables (cooked vegetables, raw vegetables), sugary drinks, and condiments (oil, butter). Frequencies derived from the food frequency questionnaire (FFQ) were compared with the Mediterranean standards [40].

The present study was approved by the local ethics committee (Study protocol: COMETA-DMD, 437/2023—DB id 13,411). To participate in the study, assent was obtained from all patients, and consent was obtained from their parents. The study was conducted in accordance with the 1964 Declaration of Helsinki and its later amendments.

### DNA Extraction and Sequencing

DNA extraction was performed using the Zymo Biomics DNA/RNA Miniprep Kit (Zymo Research), and the quality was assessed with Qubit dsDNA BR or HS (Thermo Fisher), following the manufacturer’s instructions. Twenty nanograms of DNA were utilized for library preparation by amplifying the V3-V4 hypervariable regions of the bacterial 16S rRNA gene with the Zymo Quick 16S NGS library preparation kit (Zymo Research). The final libraries were combined and sequenced on the MiSeq Sequencing System (Illumina) with 2 × 250 base paired-end reads (90.71% of bases ≥ Q30). Sequencing reads were demultiplexed using Illumina’s bcl2fastq (v2.20). Reads containing Ns or low-quality bases were removed, and adapter sequences were trimmed using Skewer (version 0.2.2) [41]. Reads merging was performed with the R package DADA2 [42], and the quality of the FASTQ files was assessed using FastQC (version 0.11.5-cegat) [43].

### Preprocessing of 16S rRNA Gene Sequences

Each pair of FASTQ files for each DMD patient was analyzed by Kraken2 using Greengenes [44] database as a reference for taxonomic classification of microbial sequences. Kraken2 is the most popular tool for taxonomic sequence classification of metagenomic sequence data. Greengenes is a curated, annotated, chimera-checked, full-length 16S rRNA gene sequences database [45].

Taxonomic assignments made by Kraken2 were then analyzed by Bracken using Greengenes as the reference database and default parameters to compute the abundance at the species level from the reads collected in a metagenomics experiment.

To integrate patient metagenomic data into one dataset, Bracken reports of all patients were loaded in Pavian [46], an interactive tool for visualizing and analyzing metagenomics data, and the resulting table was used as the reference dataset for the subsequent statistical analyses.

### Statistical Analyses

For the study sample descriptive statistics, continuous variables were shown as median and interquartile range (IQR), while nominal variables as relative and percentage frequencies. Mann–Whitney *U* and Fisher’s exact tests were applied to assess differences between DMD + and DMD − for continuous and categorical variables, respectively.

The alpha diversity, assessing the intra-community species richness of the two experimental groups, was assessed using various indicators, including the Chao Index, Simpson inverse diversity, Gini, Shannon, and Fisher diversity indices, all computed with the *alpha* function from the BioconductoR *microbiome* package [47]. Differences between DMD + vs. DMD − were analyzed using generalized linear models, adjusting for potential confounders (age, diet, steroids, ventilator support, ambulatory status, and BMI).

The beta diversity, defining the inter-community species richness between the two experimental groups, was investigated using the analysis of differential abundance taking sample variation into account (ALDEx2) tool [48], specifically designed for differential abundance analysis in microbiome datasets, particularly those derived from 16S rRNA sequencing. Operational taxonomic units (OTUs) were aggregated at various taxonomic levels: phylum, class, order, family, genus, and species. Data were transformed using a centered log-ratio (CLR) transformation before differential abundance analysis. Differential abundance was tested using a generalized linear model implemented in ALDEx2, accounting for potential confounders. False discovery rate (FDR) correction for multiple testing was applied to reduce the type I error rate.

## Results

### Population

Descriptive statistics of demographic and clinical information of the 50 DMD patients enrolled in the study are provided in Table 1.

**Table 1:** Cohort demographic and clinical information

	Total	DMD +	DMD −	*p* value
	*N* = 50	*N* = 17	*N* = 33	
Age				< 0.05
Median (Q1–Q3)	13.12 (10.15/18.80)	10.74 (6.26/13.76)	14.92 (11.58/19.23)	
On steroid treatment				ns
Y/N (%)	43 (86%)	15 (88%)	28 (84%)	
Steroid duration (years)				ns
Median (Q1–Q3)	6.83 (4.26/13.12)	4.92 (3.54/9.83)	8.51 (5.21/14.41)	
FSIQ*				< 0.001
Median (Q1–Q3)	84.00 (73.50/97.50)	56.50 (55.25/60.75)	92.00 (82.00/98.00)	
Ambulant				< 0.05
Y (%)	32 (64%)	14 (87.5%)	18 (54%)	
On NIV support				ns
Y/N (%)	10 (20%)	1 (5.88%)	9 (27.27%)	
Overweight-BMI SDS > + 2				ns
Y/N (%)	3 (5.08%)	0 (0.00%)	3 (9.09%)	
Underweight-BMI SDS < − 2				ns
Y/N (%)	6 (10.17%)	3 (17.65%)	3 (9.09%)	
Genetic grouping				ns
Group 1				
Y/N (%)	22 (44%)	4 (23.5%)	18 (54%)	
Group 2				
Y/N (%)	15 (30%	8 (47%)	7 (21%)	
Group 3				
Y/N (%)	1 (2%)	1 (5.9%)	0 (0%)	
Group other				
Y/N (%)	12 (24%)	4 (23%)	8 (24%)	
Region of origin				ns
North of Italy				
Y/N (%)	31 (62%)	12 (70%)	19 (87%)	
Center of Italy				
Y/N (%)	3 (6%)	/	3 (9%)	
South of Italy				
Y/N (%)	16 (32%)	5 (29%)	11 (33%)	

*ns*, not significant; *DMD*, Duchenne muscular dystrophy; *Q1*, first quartile; *Q3*, third quartile; *FSIQ*, full scale intelligent quotient; *NIV*, non-invasive ventilation; *BMI*, body mass index; *SDS*, standard deviation score. *FSIQ data available in 30 patients (7 DMD + and 23 DMD −). Values are expressed as median and IQR or as absolute frequency and percentage. The Kruskall-Wallis or Chi-squared test for non-parametric data has been applied

The median age was 13.1 years; 43/50 (86%) of patients were treated with daily deflazacort at a dose of 0.9 mg/kg/day, and all subjects were on supplementation with oral vitamin D at a dose of 500 UI/day.

According to genetic mutation, 22/50 patients (44%) were included in group 1 (variants upstream of exon 44 affecting Dp427 expression only), 15/50 (30%) in group 2 (variants in exons 51–62 affecting Dp427 and Dp140 expression), and 1/50 in group 3 (variants downstream of exon 63 impacting all brain isoforms Dp427, Dp14, and Dp71), while 12/50 patients (22%) were excluded from this classification. These differences were not statistically significant according to the Fisher exact test. DMD + and DMD − groups differ significantly in age and ambulatory status (Table 1). No significant differences were observed for steroids treatment, NIV support, BMI, or center of residence.

### Intellectual Function Evaluation

Thirty out of 50 patients (60%) underwent evaluation with an intelligence scale appropriate for their age, 5/50 patients (10%) had severe mental retardation, precluding the application of any scale, and 15/50 patients (30%) were not tested due to logistic constraints, being subsequently categorized into the DMD + or DMD − groups as previously described.

Accordingly, 17/50 patients (34%) were included in the DMD + group, and the FSIQ score was calculated for seven of them (median 57; IQR 55–61) (Table 1). Meanwhile, 33/50 patients (66%) were included in the DMD − group, and the FSIQ score was calculated for 21 of them (median 92; IQR 82–98) (Table 1). The IQ was significantly lower in the DMD + group (Mann–Whitney *U* test *p* value < 0.001), and overall, the FSIQ score, regardless of DMD + and DMD − grouping, was 84.

Due to the limited sample size of our cohort, and the presence of only one patient in group 3, no correlations were assessed between the genetic group and the intellectual and metagenomics profiles.

### Bristol Stool Form Scale

The BSFS was available for 35/50 patients (70%). Among them, 14/35 (40%) had a score ≤ 2, indicating a tendency toward constipation; 17/35 patients (48.6%) had a score between 3 and 4, considered normal; 4 out of 35 patients (11.4%) had a score ≥ 5, indicating a diarrheal bowel (data not shown). No significant differences between DMD + and DMD − were observed regarding the BSFS results.

### Taxonomic Analysis

The gut microbiota composition was explored by 16S rRNA analysis, and a total of 98 taxa were identified.

The alpha-diversity, assessed by several indices, did not show significant differences between DMD + and DMD − groups (Supplementary Table 1).

The beta-diversity was subsequently evaluated between the two experimental groups at the level of dominant phylum, family, and genus, after adjusting for other covariates (age, diet, steroids, NIV support, ambulatory status, and BMI) and applying FDR adjustment for multiple comparisons.

**Fig. 1:**
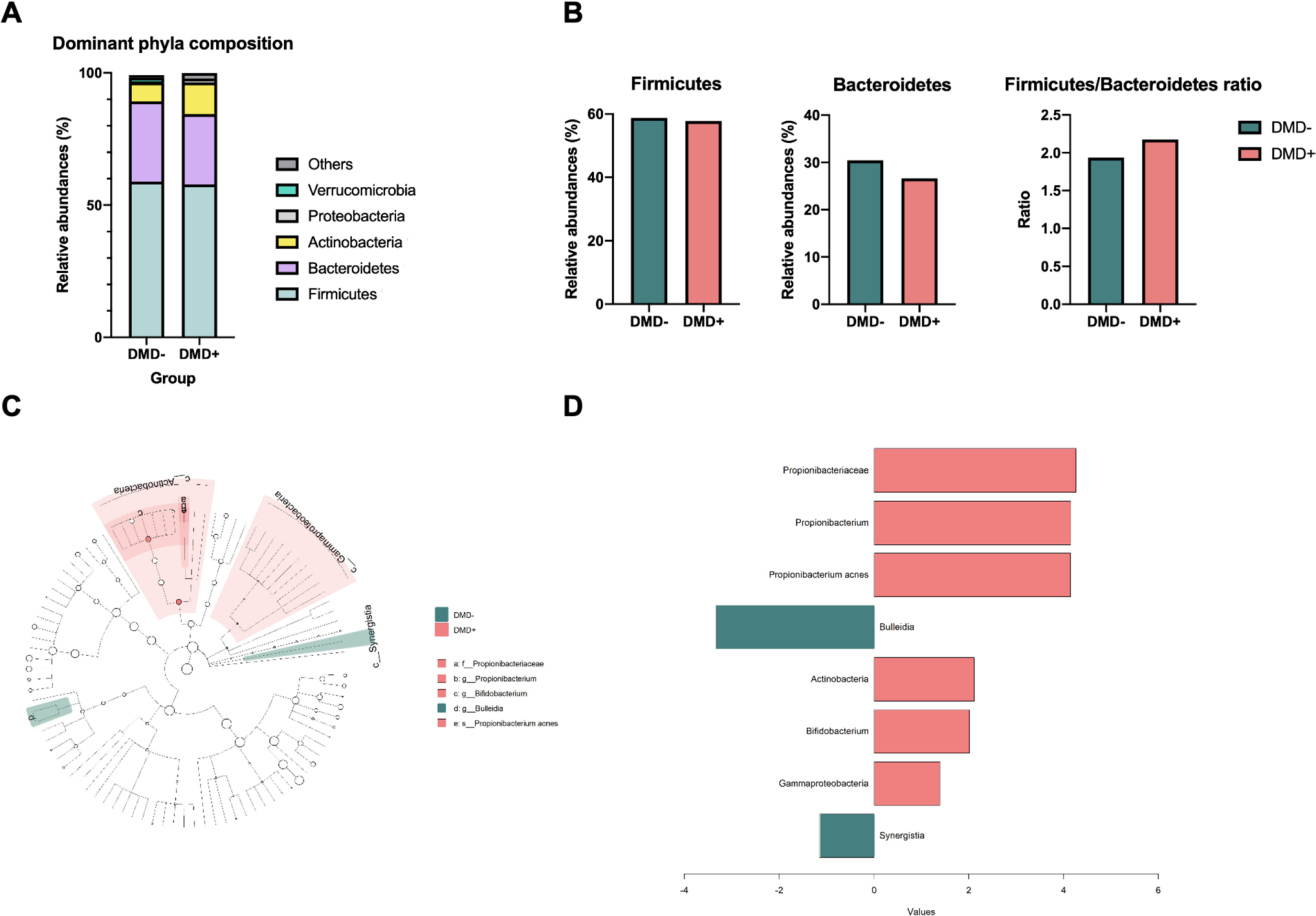
Beta-diversity analysis in DMD + vs. DMD − patients. **A** Barplots showing the dominant microbial phyla composition at the phylum level between DMD + and DMD − groups. Phyla include *Firmicutes*, *Bacteroidetes*, and *Actinobacteria*. **B** Barplots showing the relative abundance of *Firmicutes*, *Bacteroidetes*, and *Firmicutes*/*Bacteroidetes* ratio between DMD + and DMD −. A Mann–Whitney *U* test was applied to compare the two groups. **C** Cladogram generated from the ALDEx2 analysis showing significant differences in microbial features between DMD + and DMD − : features higher in DMD + are shown in red, features higher in DMD − are shown in green. **D** Fold-change barplot from ALDEx2 analysis displaying key taxa enriched in DMD + and DMD − groups

Within the six major taxonomic ranks, no differences were observed in the dominant phyla composition (Fig. 1A). The relative abundances of *Firmicutes*, *Bacteroidetes*, and the *Firmicutes*/*Bacteroidetes* (F/B) ratio, calculated based on the relative abundance of the dominant phyla *Firmicutes* and *Bacteroidetes*, were not statistically significantly different between DMD + and DMD − (Fig. 1B).

**Fig. 2:**
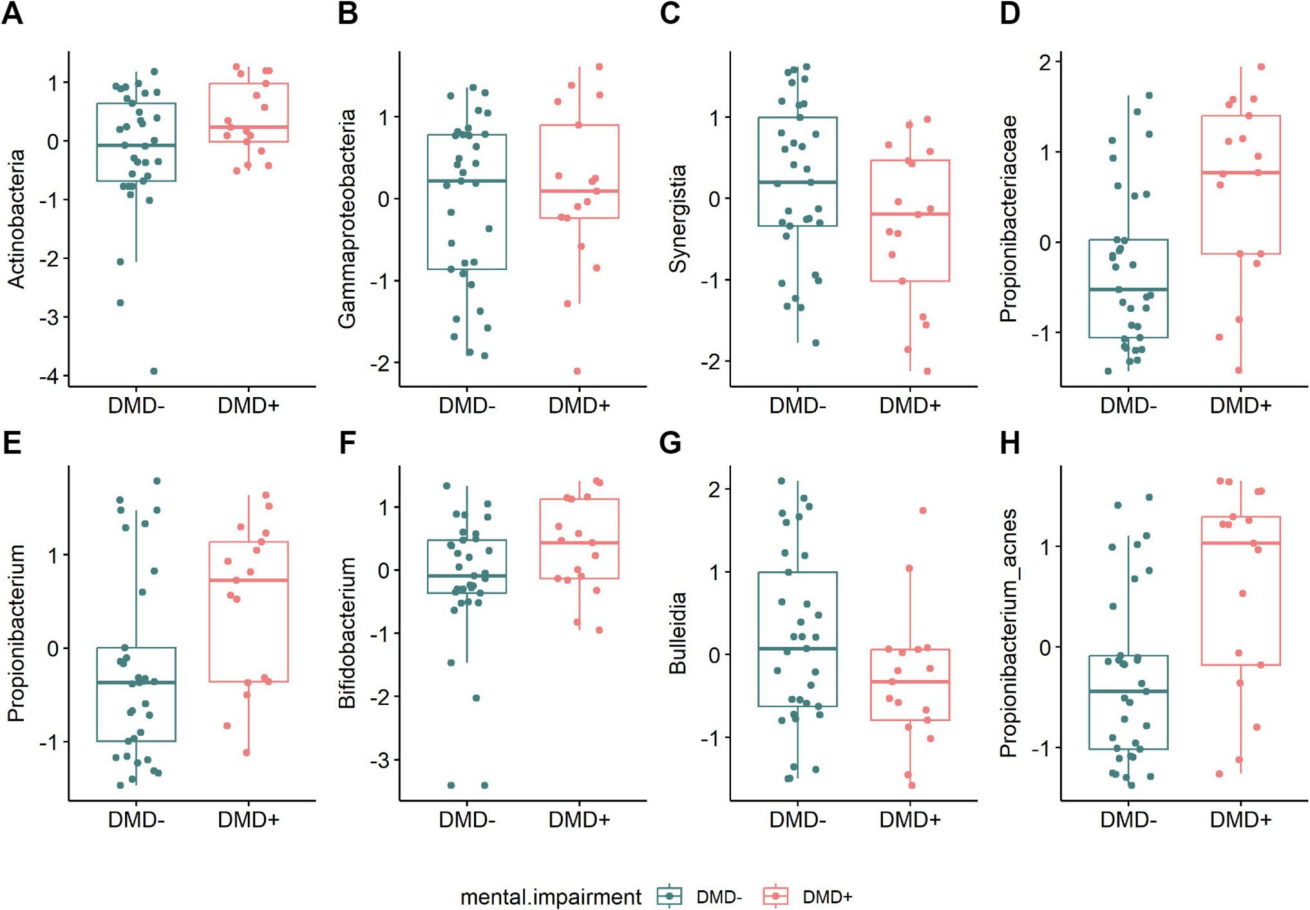
Significantly modulated taxa between DMD + and DMD − patients. **A**–**C** Boxplots showing scaled transformed abundances in DMD + vs. DMD − patients at the class, **D** family, **E** − **G** genus, and **H** species levels

However, significant differential abundances were observed between DMD + and DMD − groups (Fig. 1C–D) at the class (*Actinobacteria*, *Gammaproteobacteria*, and *Synergistia*) (Fig. 2A–C), family (*Propionibacteriaceae*) (Fig. 2D), genus (*Propionibacterium*, *Bifidobacterium*, *Bulleidia*) (Fig. 2E–G), and species (*Propionibacterium acnes*) levels (Fig. 2H; Supplementary Fig. 1A–F).

In particular, at the family level, *Propionibacteriaceae* were significantly higher in the DMD + group (Fig. 2D), and at the genus level, *Propionibacterium* (Fig. 2E) and *Bifidobacterium* (Fig. 2F), were significantly higher in DMD +, while *Bulleidia*, was lower in this group (Fig. 2G).

We investigated the association of the FSIQ score with the family *Propionibacteriaceae* (Spearman’s test, rho =  − 0.12; *p* value = 0.57) and the genera *Propionibacterium* (Spearman’s test, rho =  − 0.12; *p* value = 0.57), *Bifidobacterium* (Spearman’s test, rho = 0.12; *p* value = 0.57), and *Bulleidia* (Spearman’s test, rho = 0.26; *p* value = 0.19), without identifying any statistically significant association.

No significant differences in metagenomics profiles were detected between steroid-naïve-DMD and patients under steroid treatment.

### Nutritional Questionnaire

The weekly consumption of macronutrients, assessed with the FFQ, did not show significant differences between DMD + and DMD − patients (Supplementary Table 2). Results from the FFQ were compared to the Mediterranean standards. Overall, a deviation from these standards was observed in DMD patients, consistent with a higher consumption of red meat and ham (1.5 times), white flour (12 to 17 times), and sugary drinks (1.5 times), without any statistically significant difference between the two experimental groups (Supplementary Table 2).

## Discussion

This study aimed to assess any significant difference in the gut microbiota signature between DMD patients with and without intellectual disability, by 16S rRNA metagenomics sequencing analysis.

Thirty-four percent of the patients in our cohort showed ID and/or poor academic performance (DMD +) in line with data from the literature, and the FSIQ score, calculated disregarding the DMD + and DMD − grouping, was in the low average range [7, 8, 12].

We assessed general indexes of dysbiosis [49, 50], such as the BSFS, the alpha-diversity, and the overall abundance of the main phyla *Firmicutes* and *Bacteroidetes*, without identifying significant differences between the DMD + and DMD − groups.

However, the beta-diversity index identified a statistically significant increase in *Propionibacterium* and *Bifidobacterium* (belonging to the *Actinobacteria* phylum), and a significant reduction in *Bulleidia* (belonging to the *Firmicutes* phylum) in DMD + subjects.

*Propionibacterium* is known for its diverse metabolic capabilities, including the production of propionic acid (PPA), which, together with acetic acid (AA) and butyric acid (BA), is part of the class of enteric SCFAs [17]. While in eubiotic conditions, PPA plays a crucial role by modulating microglia development and function, and protecting the BBB [18, 51], increased PPA levels have been associated with different outcomes in autism spectrum disorders (ASDs) and depression [19, 52–54].

Significantly elevated levels of PPA or PPA-producer species have been detected in ASD patients stool samples [55–57], and PPA has been administered in animal models to evaluate the functional effects of heightened PPA levels [58–62]. Overall, these studies proved that exposure to high concentrations of PPA in rodents induces autism-like behavioral, electrographic, neuroinflammatory, and metabolic changes, resembling those observed in patients with ASDs [58–62]. The mechanisms underlying these changes are hypothesized to involve enhanced glutamate, serotonin, and dopamine release, increased sensitivity of glutamate receptors, inhibition of GABAergic receptors, mitochondrial dysfunction, closure of gap junctions, and epigenetic modifications by inhibiting the histone deacetylase (HDACI) activity [19]. Moreover, the administration of propionic acid (PPA) in rats exposed to chronic unpredictable mild stress (CUMS), a widely employed model for depression, elicited divergent and dose-dependent effects. Low doses of propionate were linked to the amelioration of depression-like symptoms, whereas high doses induced pro-depressant effects 55, and the depressive symptoms were correlated with disturbances in neurotransmitter concentrations. Collectively, these data suggest that dysregulated levels of PPA can significantly contribute to altered neurotransmitter abundance, potentially modulating neurological outcomes in ASDs and depression.

In the DMD scenario, where neurotransmitter imbalance and altered sensitivity of GABA receptors have been described in preclinical models [63–66], the higher abundance of PPA-producing *Propionibacterium* in the DMD + group represents an interesting finding. Validation in larger DMD cohorts and further functional assessment of PPA levels in stool and blood samples will be essential to clarify its potential impact on intellectual outcomes.

In addition, considering the widespread use of PPA in the food industry as a food additive [67], and the fact that the content of intestinal propionic acid can be modulated through dietary changes, probiotics, and prebiotics [68], a restriction on the consumption of ultra-processed foods among DMD + patients should be pursued to mitigate the risk of propionate overload from dietary sources. Unfortunately, our dietary questionnaire did not capture the intake of processed foods, impeding our ability to ascertain whether DMD + patients consumed more processed foods compared to the DMD- − group.

DMD + patients also exhibited augmented levels of *Bifidobacterium*. The effect of *Bifidobacterium* as a probiotic has been largely studied and found to improve gut health, to enhance immune function, and to positively modulate the gut-brain axis [69, 70]. In ASD, where a lower abundance of *Bifidobacterium* has been identified in stool samples in several studies [71, 72], *Bifidobacterium* supplementation as a probiotic produced different results. Most studies reported an amelioration of specific ASD symptoms, highlighting improvements in social behavior, communication skills, and reduction in gastrointestinal issues [73–75], although one study reported a worsening of ASD manifestations following *Bifidobacterium* supplementation [76, 77]. This suggests that, while *Bifidobacterium* holds potential therapeutic benefits, its effects may vary depending on individual differences and other underlying factors.

In addition, DMD + patients showed a lower abundance of *Bulleidia* compared to DMD −. To date, the role of *Bulleidia* in the gut-microbiota axis is largely unknown, although high levels of this genus have been detected in patients with schizophrenia [78]. Further studies are needed to elucidate the effects of *Bulleidia* in DMD and, in general, in the modulation of the gut-brain axis.

We acknowledge some limitations of the study, including the lack of a healthy control group, which limits the ability to determine if observed differences are specific to DMD with intellectual disability or general features of DMD, and the relatively small sample size for a microbiome study. Other limitations include the use of school performance as a proxy for intellectual function in some patients which could represent a bias, the lack of a complete assessment of the neurocognitive and neurobehavioral profile, the potential dietary variation among patients (even if we adjusted for dietary pattern in the main statistical analyses) as they were not put on a standard diet before enrollment, the possible effect of additional environmental factors (exposure to air pollution or other chemicals) on microbiome composition, and the lack of functional measurements of the microbiota (e.g., SCFA levels in stool or blood). Additionally, we acknowledge that multiple confounding factors—including genetic background, medication use, and gut motility—could influence our results. Although we attempted to control for these variables in our statistical models, residual confounding cannot be excluded.

Despite these limitations, while previous studies have explored the impact of dystrophin mutations on brain function, this is among the first to associate microbial changes with intellectual performances in DMD. Although we identified some microbial differences in DMD with and without ID, our study does not establish a causal relationship between gut microbiota composition and cognitive outcomes in DMD, but only suggest a correlation. We therefore highlight the need for further research integrating multi-omics approaches and longitudinal assessments which would clarify these potential interactions.

## Supplementary Information

Below is the link to the electronic supplementary material.

Supplementary file1 differences in the abundance of taxa and pathways between DMD + and DMD- patients. Volcano plots showing differences in the abundance of taxa between DMD + and DMD- patients at (A) Phylum, (B) Class, (C) Order, (D) Family, (E) Genus, and (F) Species levels. Standardized effect size between DMD + and DMD- on the x-axis; -log10(p-value) is shown on the y-axis. (PNG 322 KB)

Supplementary file2 Table 1: alpha-diversity analysis did not identify differences between DMD + and DMD- patients. Estimated differences (and 95% confidence intervals) between DMD- (reference) and DMD + patients for six alpha-diversity indices, according to linear regression models adjusted for possible confounders (age, diet, steroids, ventilator support and BMI). Table 2: weekly macronutrients consumption frequencies in DMD + and DMD-patients. Values are expressed as median and IQR or as absolute frequency and percentage. The Kruskall-Wallis test for non-parametric data has been applied. (DOCX 16 KB)

## Data Availability

Sequence data that support the findings of this study are available via the NIH Sequence Read Archive. Sequence data are available via BioProject PRJNA1169821. All other data are available from the corresponding author upon reasonable request.
