# Supplementary figures and images for: Exploratory Analysis of Gut Microbiota Profile in Duchenne Muscular Dystrophy (DMD) Patients with Intellectual Disability
Source: Mol Neurobiol. 2025 May 5;62(9):11799–809. doi: 10.1007/s12035-025-04974-7 (PMC12367937; doi:10.1007/s12035-025-04974-7)

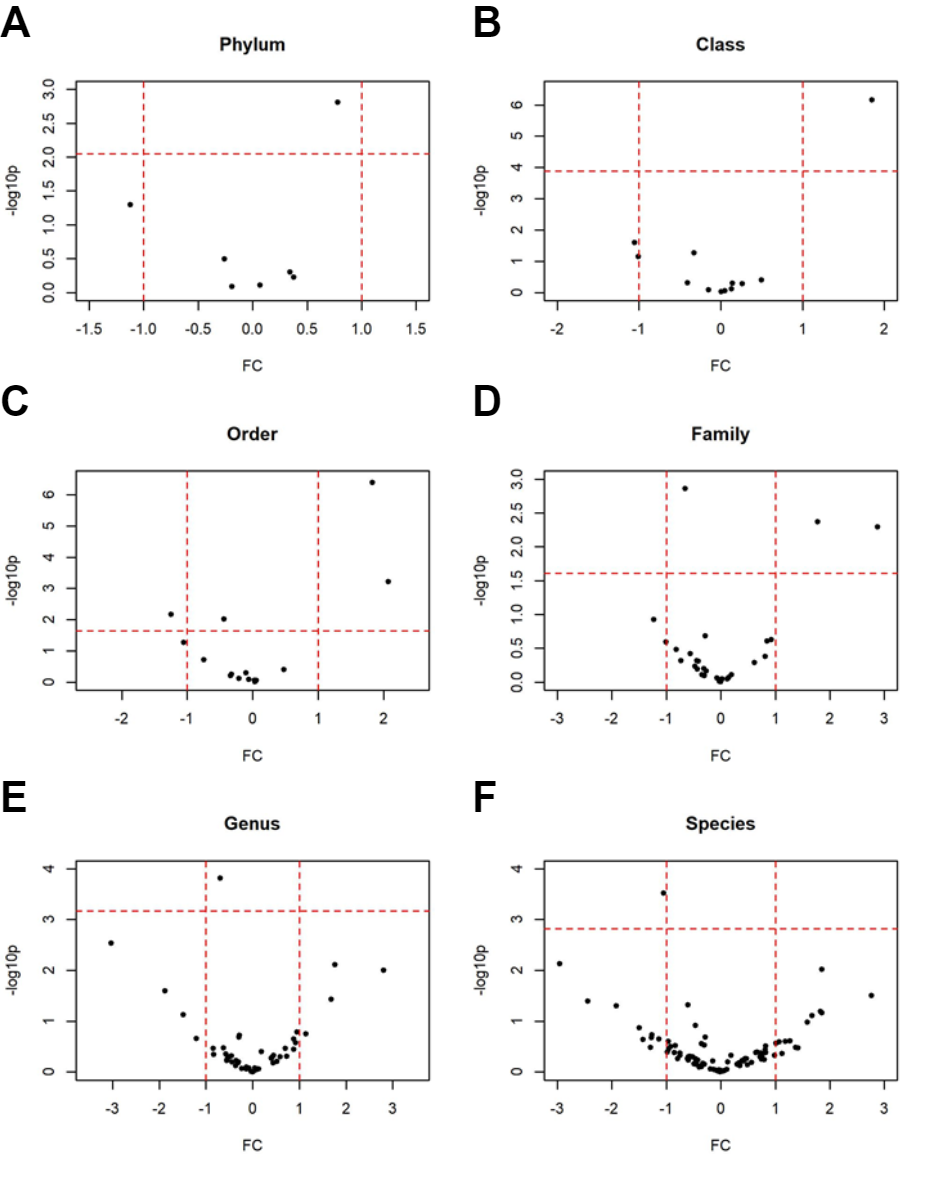

Supplement: Supplementary file 1 — Supplementary file1 differences in the abundance of taxa and pathways between DMD + and DMD- patients. Volcano plots showing differences in the abundance of taxa between DMD + and DMD- patients at (A) Phylum, (B) Class, (C) Order, (D) Family, (E) Genus, and (F) Species levels. Standardized effect size between DMD + and DMD- on the x-axis; -log10(p-value) is shown on the y-axis. (PNG 322 KB) [file 12035_2025_4974_MOESM1_ESM.png]
