# Supplementary material for: Exploratory Analysis of Gut Microbiota Profile in Duchenne Muscular Dystrophy (DMD) Patients with Intellectual Disability
Source: Mol Neurobiol. 2025 May 5;62(9):11799–809. doi: 10.1007/s12035-025-04974-7 (PMC12367937; doi:10.1007/s12035-025-04974-7)
Supplement: Supplementary file 2 — Supplementary file2 Table 1: alpha-diversity analysis did not identify differences between DMD + and DMD- patients. Estimated differences (and 95% confidence intervals) between DMD- (reference) and DMD + patients for six alpha-diversity indices, according to linear regression models adjusted for possible confounders (age, diet, steroids, ventilator support and BMI). Table 2: weekly macronutrients consumption frequencies in DMD + and DMD-patients. Values are expressed as median and IQR or as absolute frequency and percentage. The Kruskall-Wallis test for non-parametric data has been applied. (DOCX 16 KB) [file 12035_2025_4974_MOESM2_ESM.docx]

**Supplementary table 1**: **alpha-diversity analysis did not identify differences between DMD+ and DMD- patients**

| **alpha diversity index** | **estimate (95% CI)** | **p-value** |
| --- | --- | --- |
| Chao | -1.00 (-4.12; 2.12) | 0.53 |
| Inverse Simpson | 0.40 (-0.99; 1.79) | 0.57 |
| Gini-Simpson | 0.02 (-0.02; 0.06) | 0.38 |
| Shannon | 0.08 (-0.06; 0.22) | 0.29 |
| Fisher | -0.06 (0.33; 0.21) | 0.68 |
| Coverage | 0.13 (-0.44, 0.70) | 0.66 |

**Supplementary Table 2: weekly macronutrients consumption frequencies in DMD+ and DMD-patients**

|  | DMD+ (N=17) | DMD- (N=33) |  | DMD+ (N=17) | DMD- (N=33) |  |
| --- | --- | --- | --- | --- | --- | --- |
| **Macronutrients** | median (Q1/Q3) | median (Q1/Q3) | p-value | fold change to mediterranean standards | fold change to mediterranean standards | p-value |
| **Proteins** |  |  |  |  |  |  |
| **Red meat and ham** | 3 (3/5) | 3 (3/5) | ns | 1.5 | 1.5 | ns |
| **White meat and Fish** | 3 (3/5) | 3 (3/5) | ns | 0.5 | 0.83 | ns |
| **Eggs** | 1.5 (1.5/1.5) | 1.5 (1.5/1.5) | ns | 0.37 | 0.37 | ns |
| **Cheese and yogurth** | 5 (3.5/10.5) | 6 (3/10) | ns | 0.71 | 0.85 | ns |
| **Total protein** | 15 (12.5/20.5) | 16.5 (12.5/21.8) | ns | 0.78 | 0.86 | ns |
| **Carbohydrates** |  |  |  |  |  |  |
| **White flour** | 17 (10/17) | 12.75 (8.8/17) | ns | 17 | 12 | ns |
| **Whole flour** | 1.5 (1.5/3.5) | 1.5 (1.5/4.1) | ns | 0.1 | 0.1 | ns |
| **Fruits** | 7 (1.5/7) | 7 (2/7) | ns | 0.5 | 0.5 | ns |
| **Total carbohydrates** | 25.5 (20/29) | 22 (16.6/30.5) | ns | 0.9 | 0.78 | ns |
| **Vegetables** |  |  |  |  |  |  |
| **Cooked vegetables** | 3.5 (1.5/3.5) | 3.5 (1.5/3.5) | ns | 0.25 | 0.25 | ns |
| **Raw vegetables** | 1.5 (0/3.5) | 3.5 (0/6.1) | ns | 0.1 | 0.25 | ns |
| **Total vegetables** | 5 (3.5/7) | 5 (3.1/8.1) | ns | 0.35 | 0.35 | ns |
| **Sugary drinks** |  |  |  |  |  |  |
| **Sugary drinks** | 1.5 (0/7) | 1.5 (0/3.5) | ns | 1.5 | 1.5 | ns |
| **Condiments** |  |  |  |  |  |  |
| **Oil** | 7.5 (2.5/12.5) | 7.5 (2.5/7.5) | ns | 0.53 | 0.53 | ns |
| **Butter** | 0 (0/2.5) | 0 (0/2.5) | ns | 0 | 0 | ns |
| **Total condiments** | 10 (5/12.5) | 7.5 (2.5/10) | ns | 0.6 | 0.46 | ns |

Abbreviations: ns: not significant; DMD: Duchenne Muscular Dystrophy; Q1: first quartile; Q3: third quartile.
